# Worsening Hiccups, Dyspnea, and Angina in a 67-Year-Old Woman: A Challenging Case

**DOI:** 10.7759/cureus.85189

**Published:** 2025-06-01

**Authors:** Stefanos Votsis, Jaime Caballero, Cezar Iliescu, Konstantinos Marmagkiolis

**Affiliations:** 1 Cardiology, Medical College of Georgia at Augusta University, Augusta, USA; 2 Cardiology, 424 Military Hospital, Thessaloniki, GRC; 3 Cardiology, University of South Florida, Tampa, USA; 4 Department of Cardiology, Division of Internal Medicine, MD Anderson Cancer Center, Houston, USA

**Keywords:** adult cardiac surgery, angina, cardiac ct, dyspnea, left atrial appendage aneurysm

## Abstract

In this publication, we present a left atrial appendage aneurysm (LAAA) case diagnosed with a cardiac CT scan of a 67-year-old woman with worsening hiccups, dyspnea, and chest pain. This is a rare cardiac condition with only 150 cases reported to date. LAAA can manifest as diastolic dysfunction, angina, hiccups, arrhythmias, dyspnea, and rare but potentially serious complications such as systemic embolism and rupture leading to death. Our findings highlight the importance of early surgical intervention, even in asymptomatic patients, to mitigate potential thromboembolic complications and address associated atrial arrhythmias.

## Introduction

Anatomic variations of various cardiac structures are not always apparent and might prove challenging to diagnose [1]. Initial suspicion usually depends on clinical symptoms, which are oftentimes non-specific. Various cardiac imaging entities may prove extremely useful in reaching diagnosis, as well as tailoring the appropriate therapeutic management of these patients.

One of the most uncommon conditions that has garnered attention from the medical community due to its low incidence and varied clinical manifestations is the left atrial appendage aneurysm (LAAA). The difficulty in identification is reflected in its incidental detection in imaging studies such as echocardiograms (both transthoracic and transoesophageal) and tomographies (both computer and magnetic resonance), while symptoms range from mild to severe, including heart failure and thromboembolic events. The complex etiology includes congenital and acquired factors, and its management focuses on preventing complications through surgical resection, accompanied by medical strategies such as controlling heart rhythm and anticoagulation [2].

## Case presentation

**Figure 1:**
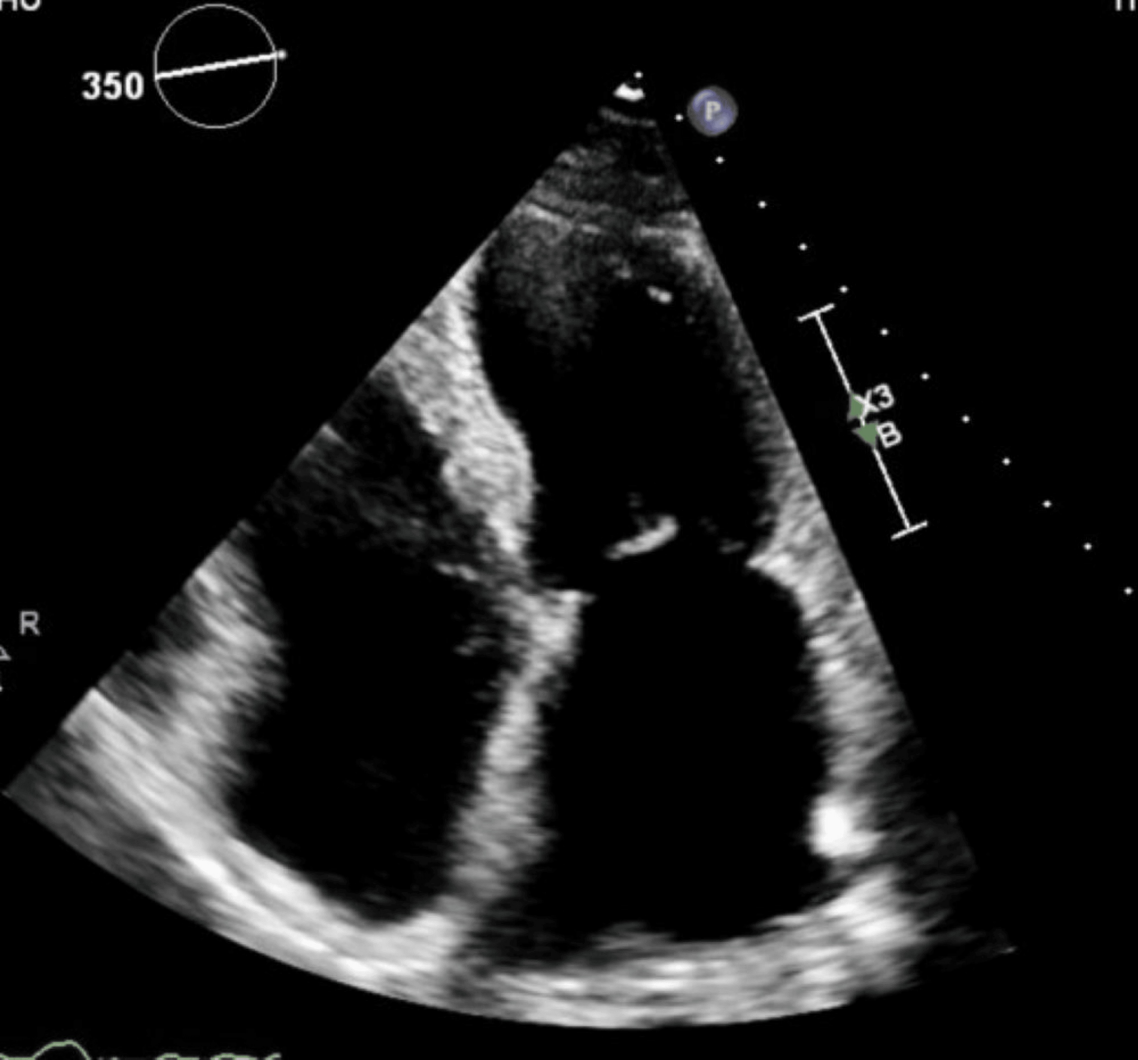
Transthoracic echocardiogram image

A patient in her mid-60s with a medical history of transient ischemic attack (TIA) and palpitations presented with worsening hiccups, dyspnea, and angina. Physical examination and bloodwork results were unremarkable. A chest X-ray, transthoracic echocardiogram (Figure 1), and a pharmacologic nuclear stress test were normal.

**Figure 2:**
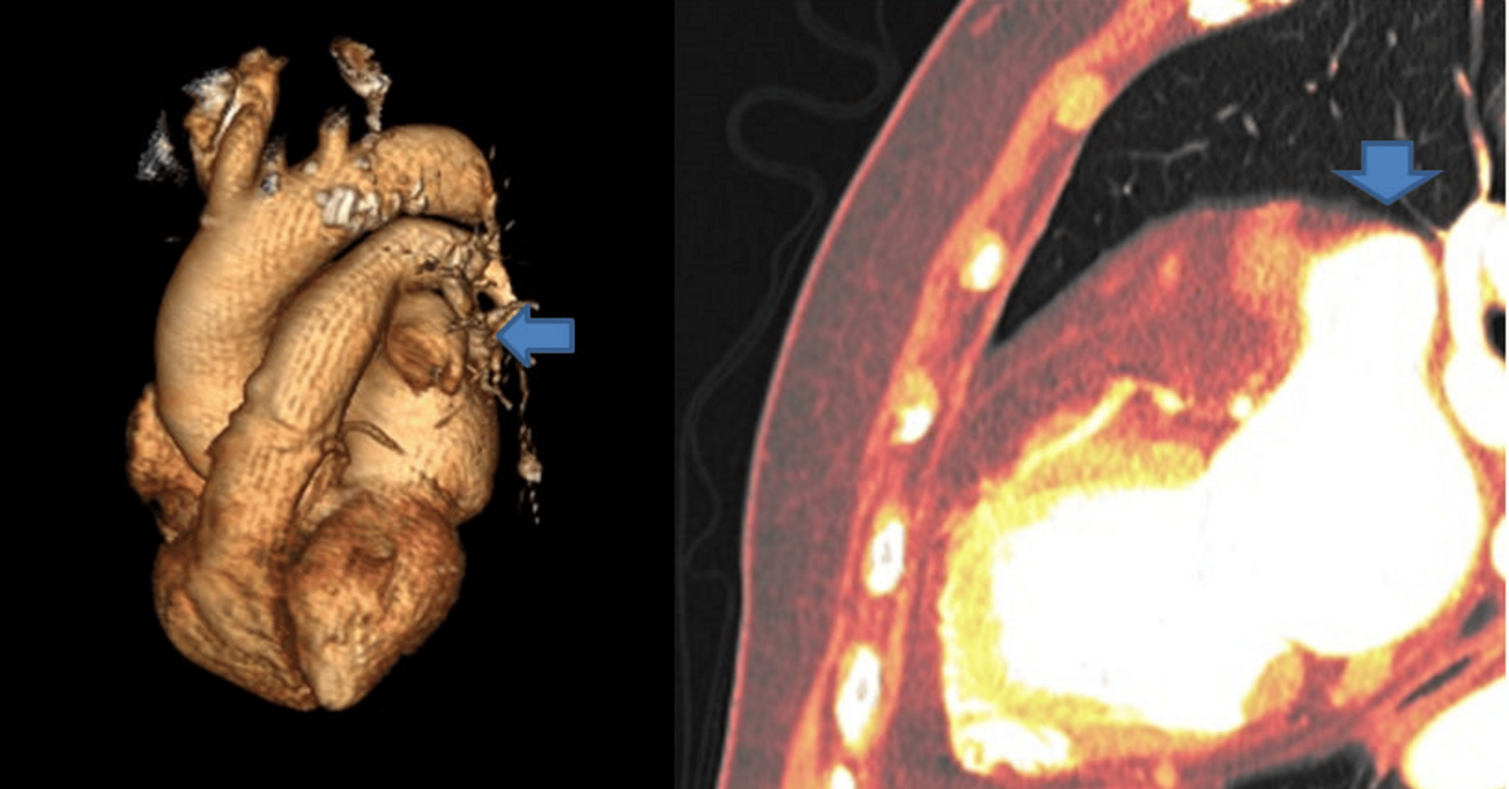
Cardiac CT 3D reconstruction image; left atrial appendage aneurysm (blue arrow)

A two-week monitor showed only rare premature ventricular contractions (PVCs). Lacking a diagnosis explaining the patient's symptoms, a further decision was made to order a cardiac CT, whose result is shown in Figure 2.

## Discussion

The cardiac CT scan demonstrates a left atrial appendage aneurysm (LAAA) (Figure 2, blue arrow) whose dimensions are measured at 6x3x3 cm, with no apparent thrombus. First documented in 1962 [3,4], approximately 150 cases have been reported in the literature to date [5]. LAAA can manifest across all age groups, with a mean age of diagnosis at 30 years. Approximately 90% of cases are congenital, associated with congenital dysplasia of the atrial pectinate muscles. The common histopathological finding is fibrosis of the endocardium or myocardium.

LAAA can lead to various clinical manifestations, including diastolic dysfunction resulting from compression of the left ventricle, angina induced by compression of the left anterior descending (LAD) coronary artery, hiccups due to irritation of the left phrenic nerve [6], arrhythmias (most commonly atrial fibrillation, atrial flutter, and supraventricular tachycardia), dyspnea accompanied by a dry, unproductive cough stemming from irritation of the respiratory tract, systemic embolism, and, in rare instances, death attributable to rupture [7].

Imaging modalities that assist the physician in the diagnosis of LAAA are chest X-ray and cardiac ultrasound (both transthoracic and transesophageal), although tomographic studies, as well as magnetic resonance imaging, play a crucial role in diagnosing and characterizing aneurysmal lesions of the cardiac cavities. Interestingly, more patients are reported to undergo cardiac CT imaging than transesophageal cardiac ultrasound (TOE) (46% vs 40%) [7], and this is also the reason for our prioritizing cardiac CT over TOE in our diagnostic algorithm.

Surgical intervention is the main treatment modality for LAAA, whether symptomatic or not, in order to prevent potential complications, and is performed in more than 75% of the patients [7]. Various effective techniques, including median sternotomy, left thoracotomy, mini-thoracotomy, and minimally invasive endoscopic approaches, have been reported for aneurysmatectomy. Our patient underwent surgical resection of the LAAA, with a favorable result.

## Conclusions

Rare clinical entities are always a part of the clinician’s diagnostic algorithm. Modern diagnostic modalities are invaluable and can prove very helpful in the resolution of the most challenging of cases.

Left atrial appendage aneurysm is one such rare clinical entity that can lead to arrhythmias and thromboembolic events. Surgical resection appears to be the primary treatment option in the current literature, and most patients show improvement or are asymptomatic after treatment. Additionally, alternative approaches, such as transcatheter closure of LAAA, ablation, and medical treatments, have been reported as viable alternatives to surgical intervention.
